# The effect of dietary patterns on mild cognitive impairment and dementia incidence among community-dwelling older adults

**DOI:** 10.3389/fnut.2022.901750

**Published:** 2022-08-08

**Authors:** Nurul Fatin Malek Rivan, Suzana Shahar, Nik Nur Izzati Nik Mohd Fakhruddin, Yee Xing You, Normah Che Din, Roslee Rajikan

**Affiliations:** ^1^Nutritional Sciences Programme, Centre for Healthy Ageing and Wellness (H-CARE), Faculty of Health Sciences, Universiti Kebangsaan Malaysia, Kuala Lumpur, Malaysia; ^2^Dietetics Programme, Centre for Healthy Ageing and Wellness (H-CARE), Faculty of Health Sciences, Universiti Kebangsaan Malaysia, Kuala Lumpur, Malaysia; ^3^Health Psychology Programme, Centre for Healthy Ageing and Wellness (H-CARE), Faculty of Health Sciences, Universiti Kebangsaan Malaysia, Kuala Lumpur, Malaysia

**Keywords:** dietary pattern, mild cognitive impairment, dementia, incidence, older adults

## Abstract

Multiple studies have shown that dietary patterns have beneficial health effects on cognitive function. However, information on this relationship is presently limited, particularly among older adults. Thus, this study aimed to determine the effects of dietary patterns on mild cognitive impairment (MCI) and dementia incidence among Malaysian community-dwelling older adults. In this prospective cohort study, a total of 280 participants aged 60 years and above were included in the 5-year follow-up analysis. Participants’ sociodemographic, medical history, anthropometry, blood pressure, body composition, biochemical indices, cognitive assessments, psychosocial functions, functional status, and dietary intake were obtained. MCI was classified based on Petersen criteria, whereas dementia status was assessed using clinical dementia rating (CDR). Univariate analysis was performed for all variables, followed by multinomial regression analysis to identify the ability of dietary patterns in predicting the incidence of MCI and dementia. After controlling for confounding factors, the findings indicated that “local snacks-fish and seafood-high salt foods” dietary pattern was associated with an increased risk of MCI incidence, where the T3 [adjusted OR = 3.943 (95% CI: 1.212–12.832), *p* = 0.032] had the highest OR compared to T2 [adjusted OR = 3.252 (95% CI: 1.108–9.546), *p* = 0.023]. Meanwhile, a negative association across the tertiles of tropical fruits-oats dietary pattern and dementia incidence was observed [T2: adjusted OR = 0.152 (95% CI: 0.026–0.871), *p* = 0.034; T3: Adjusted OR = 0.101 (95% CI: 0.011–0.967), *p* = 0.047]. In conclusion, specific dietary patterns, particularly “local snacks-fish and seafoods-high salt foods,” were shown to increase the risk of MCI, while increasing intakes of “tropical fruits-oats” dietary patterns would protect against the dementia incidence among Malaysian older adults.

## Introduction

The prevalence of age-related cognitive decline leading to dementia is rising dramatically due to the increasing aging population. To date, it is one of the most serious threats to public health worldwide (1). Mild cognitive impairment (MCI) is marked as a transition stage from a normal cognitive state to dementia, providing an opportunity to identify and prevent Alzheimer’s disease in advance (2). The prevalence of MCI ranged from 16 to 68%, while dementia was 8.5% among multi-ethnic older adults in Malaysia (3–5). Despite the high prevalence of both conditions, there is still no effective cure or pharmacological treatments to modify the course of cognitive decline and dementia in older populations. This emphasizes the crucial role of preventive strategies in reducing the incidence and slowing down the progress of neurodegenerative diseases (6). Thus, Kivipelto et al. (7) suggested that a healthy lifestyle is one of the modifiable risk factors for cognitive decline and dementia.

Diet and nutrition are the well-known modifiable risk factors for early prevention and delayed onset of age-related cognitive decline and dementia, which have been proven by many epidemiologic studies (8–10). The involvement of inflammatory mechanisms and oxidative stress in the etiology of cognitive decline and dementia reflects the crucial role of nutrition in its prevention (11). In particular, most of the previous studies in this context reported the beneficial effects of a single food or nutrient on cognitive disorders among older populations (12–14). However, the causal contribution of those nutrients and their ability in reversing the cognitive decline has remained inconclusive in the observational study and randomized controlled trials (RCTs) (13, 15). Therefore, it is recommended that meals with multiple nutrient combinations to be assessed as a cognitive decline is heterogeneous in nature and evolved from complicated processes.

Over the past years, there has been increasing interest in the effects of dietary patterns on cognition and dementia rather than a single nutrient. Dietary patterns are defined as the quantities, proportions, varieties or combinations of different foods and beverages in diets, and the frequency with which they are habitually consumed (16), enabling the analysis of potentially interactive and antagonistic effects of different nutrients. A prior study has reported that a poor nutritional status or dietary pattern is associated with faster cognitive decline and worst functional impairment among older population (17). Hence, the relationship between dietary patterns and cognition or dementia has been growing rapidly and attracted considerable attention.

Approaches to deriving dietary patterns are commonly divided into *a priori* and *a posteriori* method (18). *A priori* methods are the predefined dietary patterns created based on the nutrition guidelines or recommendations, including the Mediterranean diet and dietary approach to stop hypertension (DASH) diet. A previous study has reported that Mediterranean diet that comprises of high intake of fish, nuts, grains, olive oil, fruits, and vegetables rich in antioxidants seems to be associated with a slow rate of cognitive decline with aging (19). In addition, systematic reviews and meta-analyses have summarized the protective role of DASH and Mediterranean-DASH diet intervention in cognitive function and reducing the risk of dementia (8, 20). However, most studies were investigated among the Western population, and less is known in Asian individuals. Since food variability in different populations was based on the availability and cultural norms, these dietary patterns may not be suitable for a multi-ethnic Malaysian older adults.

*A posteriori* method is performed using multivariate statistical techniques, such as factor analysis, reduced rank regression, and cluster analysis to derive a particular dietary pattern. This approach is completely exploratory, which enable a better understanding of the study population’s existing dietary habits (18). In Malaysia, a previous local study has identified five dietary patterns through *a posteriori* method, of which the tropical fruits-oats dietary pattern was associated with successful aging (21). Also, to the best of our knowledge, no study has examined the effect of specific dietary patterns on the risk of developing mild cognitive impairment and dementia among multi-ethnic older adults. Thus, data from a population-based prospective cohort study (Long Term Research Grant Scheme-Towards Useful Ageing, LRGS-TUA) were further analyzed to determine the effects of dietary patterns on the MCI and dementia incidence among community-dwelling older adults after 5 years of follow-up.

## Materials and methods

### Study designs and participants

This longitudinal study is a follow-up of a large-scale population-based study among older adults aged 60 years and above in Malaysia (LRGS-TUA) (22) at 5-year endpoint. This study involved a multi-stage random sampling method from states with the highest prevalence of older adults representing each zone of Peninsular Malaysia; Perak (North), Selangor (Central), Kelantan (East), and Johor (South). With the assistance of the Department of Statistics Malaysia, the lists of participants were provided according to the specified areas with at least 10% of older adults (22). The inclusion criteria were Malaysian citizenship, aged 60 years and above, with no known dementia, neurological disorders, or any other psychiatric problems. The participants were excluded if they had severe vision or auditory-related difficulties and functional limitations (wheel-chair bound or bed-ridden).

**FIGURE 1:**
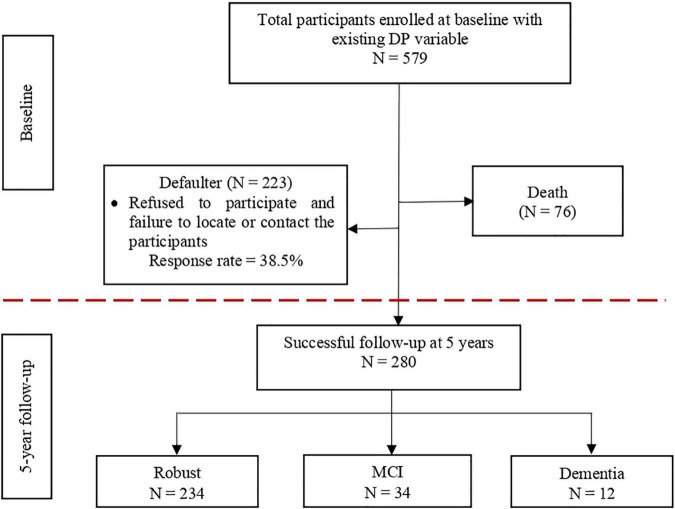
Illustration of the number of participants from the baseline to the 5-year follow-up incidence of MCI and dementia.

At baseline, a secondary analysis involving 579 out of 2,322 participants from the LRGS-TUA baseline database was conducted to analyze the dietary patterns (21). Within 5 years of follow-up, 76 participants had died, whereas 223 participants refused to participate and failed to be located or contacted (dropout rate = 38.5%). Thus, a total of 280 participants consisting of 144 men and 136 women without MCI and dementia at baseline were included in the longitudinal analysis at 5-year endpoint (Figure 1). Different fieldworkers were recruited for the follow-up assessments to comply with the blinded assessors’ criteria. This study was approved by the Medical Research and Ethics Committee of the Universiti Kebangsaan Malaysia (UKM1.21.3/244/NN-2018-145). Written information was provided, and informed written consent was obtained from all participants before they participated this study.

### Data collection

Participants were interviewed face-to-face by trained enumerators using a standardized questionnaire at their respective community centers in Malay or native language spoken by the participants. Information on sociodemographic, medical history, anthropometry, blood pressure, body composition, biochemical indices, cognitive assessments, psychosocial functions, functional status, and dietary intake of the participants was obtained. These measurements were taken at baseline and during the 5 years of follow-up period. Refreshments and honorarium were given to the participants who completed all the assessments.

After completing the data collection phase, the data cleaning process was performed and thoroughly checked to avoid any presence of missing data in the database. Each of the studied variables had less than 5% of missing data. Thus, multiple imputations of the mean score were applied to deal with missing data concerning the underlying study variables.

### Operationalization of mild cognitive impairment and dementia

The participants were characterized based on the following operationalization definition of MCI and dementia. They were categorized into three final groups after 5 years of follow-up: robust, MCI, and dementia groups.

#### Robust

Participants without MCI and dementia at baseline and after 5 years of follow-up were classified as robust.

#### Mild cognitive impairment

Mild cognitive impairment was classified based on Petersen et al. (2) and Lee et al. (23) criteria, which included preserved global function, objective memory impairment [at least 1.5 standard deviations (SD) below the mean for Rey Auditory Verbal Learning Test (RAVLT)], no limitations experienced in basic activities of daily living (ADL), had subjective memory complaint, and reported no evidence of dementia by an authorized medical officer at baseline. Subjective memory complaint by caregivers or participants was based on the question number 10 on the Geriatric Depression Scale-15 (GDS-15), where the syndrome present if the participants answered “Yes.” Preserved global function as indicated by Malay version Mini-Mental State Examination (M-MMSE) score of 19 and above (MMSE >19) was applied for this studied population (22, 24). Noted that low cutoff score was adapted in this study as these participants were older adults with low educational backgrounds.

#### Dementia

Dementia status was assessed using the clinical dementia rating (CDR) scale by an accredited researcher from health sciences background. CDR is a semi-structured clinical interview rated with a score from 0 to 3 along six dimensions, including memory, orientation, judgment, community affairs, hobbies and habits, and personal care (25). The five CDR classification categories include normal (CDR = 0), early memory decline (CDR = 0.5), and mild (CDR = 1.0), moderate (CDR = 2.0), and severe dementia (CDR = 3.0). Therefore, participants with a score of 1 and above were grouped as dementia in this study.

### Food intakes and dietary patterns

The habitual food intake of the participants was obtained using validated dietary history questionnaire (DHQ) (26). At baseline, all participants were interviewed to obtain information about their habitual food intake from wake up in the morning till before going to bed (breakfast, morning tea, lunch, afternoon tea, dinner, and supper), for the past week. Trained researchers with a degree in nutrition or dietetics conducted the interview as it required skills to extract precise information regarding their habitual food intake of the participants. The portion sizes consumed by the participants were also recorded as an indication based on the household measurement (cup, bowl, spoon, and plate) and the use of pictures from the Food Exchanges and Portion Sizes Atlas (27) to quantify the total nutrient intake. Description of cooking methods and home recipes was recorded as well. More than 350 types of food were reported and extracted from the DHQ. Fakhruddin et al. (21) classified the food intakes at baseline into 14 groups upon their similarities or references from other studies and grouped them into five dietary patterns, as indicated in Table 1. Each of the dietary patterns had a rank of tertile 1 (T1), tertile 2 (T2), and tertile 3 (T3), indicating a low, intermediate, or high intake that reflects the degree to which the individual’s diet conformed to the dietary pattern.

**TABLE 1:** Food groups based on five dietary patterns.

Dietary patterns	Food groups	Definition
**Sweet foods-beverages**	**Sweet foods:** Sweetened creamer, granulated sugar, egg jam, and fruit jam. **Beverages:** Coffee, tea, and malted drink.	A high loading of sweet foods including sugar, condensed milk, sweet spread, and beverages such as tea, coffee, and malted drink, as well as low intake of dairy products including low fat milk and butter.
**Meat-vegetables-rice and noodles**	**Meats:** Beef and pork. **Vegetables:** Brinjal, okra, carrot, cucumber, broccoli, long bean, chili, spinach, mustard leaves, tapioca shoots, *ulam raja*^a^, swamp cabbage, fern shoots, mung bean, cabbage, and kale. **Rice and noodles:** Rice porridge, white rice, dried noodle, and wet noodle.	A high intake of meat, including beef and pork, various types of vegetables and also rice and noodles.
**Local snacks-fish and seafoods-high-salt food**	**Local snacks:** Banana fritters, *cucur*^b^, banana ball, curry puff, and *cakoi*^c^. **Fish and seafood:** Pomfret, tuna, Indian mackerel, round scad, red snapper, hairtail scad, trevally, sea perch, Spanish mackerel, cuttlefish, and prawn. **High salt foods:** Salted fish, anchovy sauce, fermented durian, and chili shrimp paste.	High consumption of Malaysian snacks, fish and seafood, sauces, and condiments.
**Fruits-legumes**	**Fruits:** Red apple, green apple, and grapes. **Legumes:** Soy bean fermented cake, soy bean cake, and yellow dhal.	A high intake of a wide range of fruits other than tropical fruits, and also legumes and legume-based products.
**Tropical fruits-oats**	**Tropical fruits:** Papaya, orange, banana, duku, and rambutan. **Oats:** Oats.	A high loading of a wide range of tropical fruits that included papaya, orange, duku, banana, and rambutan and oats.

^a^Malay traditional salad.

^b^Fried bite-sized snack usually made from flour and anchovies.

^c^Long golden-brown deep-fried strip of dough commonly served with egg jam.

Source: Fakhruddin et al. (21).

### Potential confounding factors

Many factors were taken into accounts, such as sociodemographic factors, medical history, anthropometry, blood pressure, body composition, biochemical indices, and a psychosocial and functional status which might influence MCI and dementia incidence. The details of each protocol were previously published by Shahar et al. (22), including:

#### Sociodemographic information and medical history

The sociodemographic information included age, gender, years of education, ethnicity, marital status, living arrangement, household income, smoking status, and alcohol intake was obtained. Self-reported information on certain diseases (hypertension, hypercholesterolemia, diabetes mellitus, osteoarthritis, and heart diseases) diagnosed by a doctor in the prior years was recorded.

#### Anthropometry, blood pressure, and body composition

Anthropometric measurements were assessed according to the standard protocols (22), which includes weight, height, mid-upper arm circumference (MUAC), waist circumference, hip circumference, and calf circumference. Body circumferences were measured using a non-extensible and flexible plastic measuring tape. All measurements were taken two times by the same assessor to obtain reliable results. The body mass index (BMI, kg/m^2^) was calculated as the body weight in kilograms divided by the squared standing height in meters. Body composition was assessed using Bio-electrical Impedance Analysis (BIA) InBody S10 (Bio-space Co., Ltd., Seoul, South Korea). Systolic and diastolic blood pressure were also measured using a calibrated digital automatic blood pressure monitor (OMRON, Kyoto, Japan).

#### Biochemical indices

About 20 ml of fasting peripheral venous blood was withdrawn using a butterfly syringe by a trained phlebotomist for the biochemical analysis, which consisted of fasting blood sugar (FBS), total cholesterol (TC), high-density lipoprotein (HDL), low-density lipoprotein (LDL), triglyceride (TG), and albumin (ALB). The collected blood was immediately sent to the nearest clinical laboratory (PathLab™ Malaysia Sdn Bhd) for blood analysis.

#### Psychosocial and functional status

Geriatric Depression Scale-15 was used to assess potential depressive symptoms (28); Medical Outcome Study Social Support Survey (MOSS) was used to assess social support; functional status was assessed using ADL (29) and instrumental activities of daily living (IADL) (30).

#### Cognitive function test

Global cognitive function was measured using the M-MMSE. The 11-items questionnaire included questions on orientation to time and place, registration, attention and calculation, recall, language, and visual construction. A validated Malay version of MMSE (MMSE-7) by Ibrahim et al. (24) was used in this study due to the native language and cultural differences. The MMSE score was classified as follows: (1) severe cognitive impairment/dementia (MMSE <9 points); (2) moderate cognitive impairment (MMSE 10–18 points); and (3) normal cognition (MMSE >19 points) (22, 24). Meanwhile, verbal learning and memory were assessed using the RAVLT test (31). The participants were given detailed instructions on the method of administration before the test. They were required to listen carefully to the words uttered by the examiner. After each presentation, the participants needed to recall the words listed by the examiner, with no restrictions in the output order. Following that, a single-attempt free recall was conducted after presenting a new list of 15 words in List B (Trial B). Immediately after this, the examiner instructed the participant to recall words from the list [Trial A6 (immediate recall)] without rereading it. Each successful recall in any of the 15 words was given one point. The RAVLT involved calculating z-score by subtracting the participant’s raw score with the normative group mean and dividing the result with the normative group standard deviation derived from the study by Tierney et al. (32).

### Statistical analysis

Statistical analysis was performed using the Statistical Package for the Social Sciences (SPSS) IBM version 25.0 (Licensed materials – Property of SPSS Incorporation an IBM Company Copyright 1989 and 2010 SPSS Inc., Chicago, IL, United States). Descriptive data were used to obtain frequency and the percentage of the baseline attributes of the participants. Normality test was performed using the Shapiro–Wilk test. One-way ANOVA test was performed to identify the significant differences between the continuous variables and the cognitive status (robust, MCI, and dementia), whereas the Fisher’s exact test was used for the categorical variables. As mentioned previously, the dietary pattern analysis has been conducted in the previous study (21) using principal component analysis (PCA). There are five specific dietary patterns extracted based on the types of food exhibiting the strongest correlation with the highest loading factors. The factor scores of each factor were ranked as tertile groups to indicate a low, medium, or high intakes (T1, T2, and T3) of each dietary pattern.

Univariate analysis was followed by multinomial regression analysis to assess the relationship between the participants’ dietary patterns and cognitive status after 5 years of follow-up. The highest tertile was then compared with the lowest tertile of factor scores for each dietary pattern. The dependent variable was the cognitive status with a robust group as the reference variable (0–robust, 1–MCI, 2–dementia) compared to MCI and dementia. The adjusted odd ratios (adj ORs) for MCI and dementia incidence and their 95% confidence intervals (CIs) were estimated after adjusting for age, ethnicity, strata, years of education, hypertension, diabetes mellitus, MUAC, waist circumference, waist circumference, fasting blood sugar, GDS, and IADL. Significant value was set at *p* < 0.05.

## Results

After 5 years of follow-up, the majority of the participants are in the robust category (83.6%), followed by MCI (12.1%) and dementia (4.3%) among those without MCI and dementia at baseline. As stated in Table 2, the mean age of participants in this study was 67.34 ± 5.00 years old and those in the dementia group was the oldest (70.00 ± 5.41 years old) as compared to MCI (67.50 ± 4.55 years old) and robust group (67.18 ± 5.02 years old) (*p* > 0.05). About half of the participants were men (51.4%), comprised of Malay (56.1%), Chinese (40.4%), and Indians (3.6%). Majority were married (76.1%), and living together with their families (90.7%). The lowest mean of the formal education years was observed among the dementia group (3.08 ± 4.08 years), followed by MCI (3.94 ± 3.81 years) and the robust group (6.31 ± 4.05 years) (*p* < 0.05). With respect to medical history, participants in dementia group had the highest prevalence of hypertension (58.3%) and diabetes mellitus (33.3%) compared to the MCI and robust group (*p* < 0.05).

**TABLE 2:** Participant characteristics by tertile of the dietary pattern at baseline [presented as mean ± standard deviation (SD) or *n* (%)].

Characteristic	Total (*n* = 280)	Robust (*n* = 234)	MCI (*n* = 34)	Dementia (*n* = 12)	*P*-value
Age (years)	67.34 ± 5.00	67.18 ± 5.02	67.50 ± 4.55	70.00 ± 5.41	0.160
Malay	157 (56.1)	140 (59.8)	11 (32.4)	6 (50.0)	0.010*
Men	144 (51.4)	124 (53.0)	16 (47.1)	4 (33.3)	0.357
Single/not married	67 (23.9)	55 (23.5)	9 (26.5)	3 (25.0)	0.927
Living alone	26 (9.3)	20 (8.5)	4 (11.8)	2 (16.7)	0.556
Years of education	5.89 ± 4.13	6.31 ± 4.05	3.94 ± 3.81	3.08 ± 4.08	<0.001*
Household income, RM	1448.33 ± 1899.34	1525.18 ± 2007.52	1133.53 ± 1197.68	841.67 ± 998.60	0.282
Strata – Urban	163 (58.2)	130 (55.6)	26 (76.5)	7 (58.3)	0.069
Smoking status	71 (25.4)	63 (26.9)	7 (20.6)	1 (8.3)	0.280
Alcohol intake	14 (5.0)	10 (4.3)	3 (8.8)	1 (8.3)	0.452
Hypertension	86 (30.7)	64 (27.4)	15 (44.1)	7 (58.3)	0.020*
Hypercholesterolemia	63 (22.5)	46 (19.7)	13 (38.2)	4 (33.3)	0.056
Diabetes mellitus	39 (13.9)	28 (12.0)	7 (20.6)	4 (33.3)	0.046*
Osteoarthritis	27 (9.6)	24 (10.3)	1 (2.9)	2 (16.7)	0.282
Heart diseases	15 (5.4)	12 (5.1)	3 (8.8)	0 (0.0)	0.470
BMI (kg/m^2^)	24.95 ± 3.87	24.82 ± 3.83	26.17 ± 3.80	24.08 ± 4.52	0.120
MUAC (cm)	28.62 ± 2.96	28.52 ± 2.89	29.70 ± 3.19	27.33 ± 3.16	0.029*
Waist circumference (cm)	87.29 ± 10.06	86.70 ± 9.87	91.43 ± 10.14	86.88 ± 11.75	0.037*
Hip circumference (cm)	96.43 ± 8.08	98.85 ± 8.96	96.20 ± 7.84	94.03 ± 9.46	0.116
Calf circumference (cm)	33.86 ± 3.29	34.78 ± 3.49	33.82 ± 3.23	31.88 ± 3.23	0.029*
SBP (mmHg)	135.39 ± 20.12	134.35 ± 19.20	139.47 ± 21.92	144.12 ± 29.30	0.118
DBP (mmHg)	76.52 ± 11.88	76.05 ± 11.39	79.97 ± 14.22	75.95 ± 13.49	0.196
FBS (mmol/l)	5.82 ± 1.49	5.77 ± 1.27	5.74 ± 1.33	6.91 ± 3.93	0.033*
Total cholesterol (mmol/l)	5.44 ± 1.02	5.47 ± 1.02	5.21 ± 1.01	5.53 ± 0.89	0.356
HDL cholesterol (mmol/l)	1.43 ± 0.32	1.44 ± 0.31	1.40 ± 0.37	1.39 ± 0.23	0.702
LDL cholesterol (mmol/l)	3.38 ± 0.97	3.41 ± 0.97	3.12 ± 0.96	3.44 ± 0.82	0.256
Triglyceride (mmol/l)	1.40 ± 0.64	1.38 ± 0.65	1.50 ± 0.61	1.54 ± 0.58	0.428
Albumin (g/l)	42.79 ± 2.26	42.69 ± 2.08	43.53 ± 3.34	42.74 ± 1.63	0.129
Body fat (%)	39.11 ± 11.23	38.69 ± 11.12	42.28 ± 11.62	38.28 ± 11.74	0.213
SMM (kg)	19.84 ± 4.66	19.86 ± 4.54	20.40 ± 5.62	17.83 ± 3.60	0.256
GDS	2.30 ± 2.07	2.14 ± 2.02	2.92 ± 2.35	3.15 ± 2.19	0.017*
IADL	13.28 ± 1.29	13.35 ± 1.16	13.12 ± 1.61	12.25 ± 2.09	0.011*
MOSS	39.45 ± 14.85	39.96 ± 14.79	37.06 ± 15.20	36.33 ± 15.18	0.432

*p < 0.05, significant using independent t-test for continuous variables and chi-square test for categorical variables.

RM, ringgit Malaysia; BMI, body mass index; MUAC, mid-upper arm circumference; SBP, systolic blood pressure; DBP, diastolic blood pressure; FBS, fasting blood pressure; HDL, high-density lipoprotein; LDL, low-density lipoprotein; SMM, skeletal muscle mass; GDS, Geriatric Depression Scale; IADL, instrumental activity of daily living; MOSS, Medical Outcomes Study Social Support Survey; MCI, mild cognitive impairment.

The mean of MUAC of all participants was 28.62 ± 2.96 cm, whereby the participants in the dementia group had the lowest MUAC (27.33 ± 3.16 cm), followed by robust (28.52 ± 2.89 cm) and MCI group (29.70 ± 3.19 cm) (*p* < 0.05). Similarly, the calf circumference of the dementia group (31.88 ± 3.23 cm) was significantly lower than the MCI (33.82 ± 3.23) and robust group (34.78 ± 3.49 cm) (*p* < 0.05). There was a significant difference in the waist circumference with a mean of 87.29 ± 10.06 cm (*p* < 0.05). The FBS level was significantly the highest in dementia group (6.91 ± 3.93 mmol/l), followed by MCI (5.74 ± 1.33 mmol/l) and robust group (5.77 ± 1.27 mmol/l) (*p* < 0.05). Those in dementia group had the highest score of GDS (3.15 ± 2.19) and the lowest in the IADL scale (12.25 ± 2.09) compared to the MCI and robust group (*p* < 0.05), indicating that they had depressive symptoms and poor functional status.

Multinomial regression models were used to analyze the association between the dietary patterns with MCI and dementia incidence (Table 3). The analysis was adjusted for age, ethnicity, strata, years of education, hypertension, diabetes mellitus, mid-upper arm circumference, waist circumference, calf circumference, fasting blood glucose, geriatric depression symptoms, and instrumental activities of daily living. There was a positive association across the tertiles of local snacks-fish and seafoods-high salt foods dietary pattern and MCI incidence, where the T3 [adjusted OR = 3.943 (95% CI: 1.212–12.832), *p* = 0.023] had the highest OR compared to T2 [adjusted OR = 3.252 (95% CI: 1.108–9.546), *p* = 0.032]. On the contrary, a negative association across the tertiles of tropical fruits-oats dietary pattern and dementia incidence was observed [T2: adjusted OR = 0.152 (95% CI: 0.026–0.871), *p* = 0.034; T3: adjusted OR = 0.101 (95% CI: 0.011–0.967), *p* = 0.047].

**TABLE 3:** Adjusted odd ratios for MCI and dementia according to the tertiles of dietary pattern scores (*n* = 280).

Dietary patterns (Tertiles)	MCI vs. robust			Dementia vs. robust		
	*B*	Adj OR (95% CI)	*P*-value	*B*	Adj OR (95% CI)	*P*-value
Sweet food-beverages						
T1	(reference)			(reference)		
T2	0.455	1.576 (0.631–3.934)	0.330	0.083	1.086 (0.198–5.968)	0.924
T3	0.064	1.066 (0.329–3.455)	0.915	0.627	1.872 (0.325–10.789)	0.483
Meat-vegetables-rice and noodles						
T1	(reference)			(reference)		
T2	−0.339	0.712 (0.258–1.966)	0.512	1.608	4.993 (0.432–57.662)	0.198
T3	−0.527	0.591 (0.219–1.590)	0.297	2.349	10.478 (0.931–117.939)	0.057
Local snacks-fish and seafoods-high salt foods						
T1	(reference)			(reference)		
T2	1.179	3.252 (1.108–9.546)	0.032*	−1.038	0.354 (0.034–3.661)	0.384
T3	1.372	3.943 (1.212–12.832)	0.023*	1.099	3.003 (0.479–18.836)	0.241
Fruits-legumes						
T1	(reference)			(reference)		
T2	−0.457	0.633 (0.226–1.776)	0.385	0.488	1.629 (0.234–11.363)	0.622
T3	−0.530	0.588 (0.210–1.650)	0.314	1.349	3.852 (0.545–27.242)	0.177
Tropical fruits-oats						
T1	(reference)			(reference)		
T2	1.022	2.778 (0.933–8.277)	0.067	−1.886	0.152 (0.026–0.871)	0.034*
T3	0.547	1.728 (0.568–5.258)	0.335	−2.293	0.101 (0.011–0.967)	0.047*

*p < 0.05, significant using multinomial regression analysis.

Adjusted for age, ethnicity, years of education, strata, hypertension, diabetes mellitus, mid-upper arm circumference, waist circumference, calf circumference, glucose, geriatric depression symptoms, and instrumental activities of daily living. T1, T2, and T3 represent individuals in the lowest, intermediate, and highest category of the dietary factor score. Adj OR, adjusted odd ratio; CI, confidence interval; T, tertile; MCI, mild cognitive impairment.

## Discussion

In this study, high consumption of unhealthy Malaysian snacks, fish and seafood, sauces, and condiments was associated with an increased risk of MCI incidence by four times even after adjusting for potential confounders. Institute for Public Health Malaysia (33) has stated that light soy sauce, dark soy sauce, oyster sauce, and tomato and chili sauce were identified as among the top ten sodium sources in the Malaysian diet leading to high salt consumption. These high salt foods could lead to the development of cerebrovascular diseases and cognitive impairment (34). The findings from magnetic resonance imaging (MRI) study suggested that excessive salt intake may promote cerebral small vessel disease by increasing the white matter hyperintensities (35), leading to the cognitive impairment and Alzheimer’s disease (36). Dietary salt intake may also impair cognitive function independently of its strong association with blood pressure, suggesting that effects of salt on cognition might be mediated by changes to vascular function (37). Therefore, these findings suggest that lowering food with high salt contents could effectively reduce the risk of cognitive impairment in older adults.

Moreover, the findings also showed that fish and seafood were associated with an increased risk of MCI among older adults in Malaysia, which contradicts with the previous studies (38). Fish and seafood had high concentrations of omega-3 polyunsaturated fatty acids (PUFAs) such as docosahexaenoic acid (DHA) and eicosapentaenoic acid (EPA), which is beneficial for cognitive health through reducing inflammation and oxidative stress in the central nervous system (39–41). However, a meta-analysis by Zeng et al. (42) found no statistically significant association between fish intake and the risk of MCI. The inconsistencies in the findings could be due to the disparities in types of fish and the cooking methods. The most preferred cooking style among the Malaysian population was deep-fried [Ahmad et al. (43)]. Excessive heat during preparation may further reduce DHA and EPA by breaking down the double bonds for oxidation (44). Frying also promotes the production of dietary advanced glycation end products (AGEs), which is associated with cognitive impairment (45, 46).

In addition, Malaysian local snacks, including banana fritters, *cucur*, banana ball, curry puff, and *cakoi*, were also prepared by deep-frying. Overconsumption of fried foods could increase the risk of inflammation-induced diseases such as high blood pressure and atherosclerosis, which is associated with cognitive decline and dementia (47, 48). Besides that, these local snacks are often fried using reused cooking oil, inhibiting the activity of paraoxonase enzyme and causing an accumulation of low-density lipoprotein (LDL) cholesterol, which contributes to the pathogenesis of atherosclerosis and worse cognitive performance (49, 50). Reusing the oils many times under high temperature increases the formation of trans fatty acids, further contributing to the neurodegenerative diseases (51, 52). Thus, reducing the consumption of fried foods and changing the cooking methods could potentially reduce the risk of cognitive impairment in later life.

Furthermore, this study showed that the tropical fruits-oats dietary pattern has an 84.8–89.9% lower risk of dementia among Malaysian older adults. This finding was in line with other studies that reported the benefits of fruit consumption in reducing cognitive impairment and dementia (53, 54). In addition, adherence to a single component of the Mediterranean diet, a diet rich in fruits and vegetables, is associated with reduced dementia risk and slower cognitive decline (55). The anti-inflammatory properties in fruits might be beneficial in neuroprotection by reducing the inflammation due to their ability to decrease neutrophils migration to the inflammatory site and block oxidative stress (56). Tropical fruits are rich sources of natural antioxidants such as phenolic and polyphenolic compounds, flavonoids, and ascorbic acid as compared to temperate climate fruits (57). Oxidative stress and inflammation could contribute to the development and progression of dementia (58), and thus, low consumption of fruits could decrease resiliency against the neurodegenerative processes. It should be noted that increasing fruit consumption could contribute to hyperglycemia among older adults with diabetes mellitus. According to the Clinical Practice Guideline (59), patients with diabetes were recommended to take two servings of fruits with high dietary fiber and low glycemic index per day to have good glycemic control. The tropical fruits that consumed by our local population such as banana, guava, jackfruit, papaya, dragon fruit, and mango are high in dietary fiber and contain low to moderate glycemic index (GI 30.5–63.5), which could improve glycemic control of older adults with diabetes (60, 61). Thus, increasing the tropical fruit intakes based on the recommended serving size could eventually protect diabetic older adults against cognitive impairment. Besides that, oats were also found to be protective against dementia incidence among older adults. Oat has plenty of health benefits associated with dietary fibers such as β-glucan, functional protein, lipid, and starch components (62). β-glucans in oat are resistant to digestion and absorption in the small intestine, attenuating blood cholesterol, which is also associated with lowering the risk of Alzheimer’s disease. The high lipids in the brain might influence the function of cleavage enzymes such as β-secretase and γ-secretase, promoting Aβ protein production, a leading cause of Alzheimer’s disease (63). Oat also exhibits strong antioxidant activity and anti-inflammatory properties that may prevent or limit cellular oxidative dysfunctions and protect against oxidative stress-related diseases, including dementia (64). Hence, owing to the high nutritional value of this dietary pattern, this study suggests that the increase in fruit and oat consumption might possess a significant role in combating neurodegenerative disease among Malaysian older adults.

Accordingly, local snacks-fish and seafoods-high salt foods show the strongest effects on MCI, whereas tropical fruits-oats affect cognitive health status in the dementia group. The distinct effect that underlies this relationship could be due to different preferences in food choices among those with MCI and dementia older adults. Since most demented older adults live together with their family members, the meals are commonly prepared by their carer to ensure they continue to enjoy the food based on their eating behavior, such as craving for sweets or carbohydrates (65). Older adults with dementia were much older than the MCI, where poor dentition may be the cause for poor fruit and oat intake due to difficulty in chewing (66). With aging, a decrease in olfactory and gustatory functions also may alter the food preferences and thereby their dietary patterns compared to their younger counterparts (67).

Despite the significant associations between tropical fruits-oats and dementia incidence, the fruits-legumes dietary pattern shows the opposite effects. As reported by a prior study, the non-significant effects of fruits consumption on cognitive performance could be due to the measurement limitations, and the types or amounts of fruit consumption (68). Furthermore, a meta-analysis indicated that most included studies combined fruits and vegetables as the exposure variable to reduced risk of cognitive impairment and dementia (53). Low statistical power was observed in the studies that estimate the effect of fruits alone. Although many studies have provided evidence on the protective effects of legumes on cognitive decline, the presence of sensory losses due to age among the older population might compensate for the lack of interest in this type of food (69, 70). Besides, a previous study has encouraged increasing the legumes intake to three servings of legumes per week for a protective effect against cognitive decline (70). Thus, this indicates that the amount of legumes intake among our older population could be lower than the recommended as no effect was seen in this study.

There are several strengths in this study. This study is the first longitudinal study report on the effects of dietary patterns on the incidence of MCI and dementia among older adults in Malaysia. This study involved a wide range of parameters covering several domains such as sociodemographic, medical history, anthropometry, blood pressure, body composition, biochemical indices, cognitive assessments, psychosocial functions, functional status, and dietary intake that may identify the predisposing confounding factors of these adverse outcomes using simple yet valid tools. These findings also could add valuable insights into the importance of specific types of foods in alleviating neurodegenerative diseases. However, few limitations in this study should be acknowledged when interpreting the findings. First, the dietary pattern was obtained from the dietary history questionnaire (DHQ), of which the reliability and validity of dietary intakes may be impacted by memory problem, memory loss, or cognitive decline, leading to under-or over-reporting of the intakes. Second, this study used previous databases to obtain baseline information of the participants, whereby missing data on target variables were the potential limitation. Thus, multiple imputations were applied in the analysis to reduce biased estimates. Besides that, the CDR assessments were only conducted during 5 years of follow-up, thus limiting the use of this instrument for the MCI classification. Third, the high dropout rate of participants at the 5-year follow-up could contribute to under-representing the study population. However, this is a common problem in longitudinal studies involving older adults. Therefore, it is suggested that future longitudinal studies should be conducted with a larger sample size and middle-aged adults (40–59 years old) to reduce the dropout rate.

## Conclusion

In conclusion, specific dietary patterns, particularly “local snacks-fish and seafoods-high salt foods,” were shown to have an association with an increased risk of MCI. In contrast, increasing intakes of “tropical fruits-oats” dietary patterns could protect against dementia among Malaysian older adults. These findings could provide the foundation for developing dietary guidelines to improve the cognitive health of our local population. Accordingly, further research involving intervention trials of sufficient sample size is necessary to confirm whether the dietary patterns are favorable with respect to the prevention of cognitive impairment and dementia among older adults in Malaysia.

## Data availability statement

The raw data supporting the conclusions of this article will be made available by the authors, without undue reservation.

## Ethics statement

The studies involving human participants were reviewed and approved by the Medical Research and Ethics Committee of the Universiti Kebangsaan Malaysia (UKM1.21.3/244/NN-2018-145). The patients/participants provided their written informed consent to participate in this study. Written informed consent was obtained from the individual(s) for the publication of any potentially identifiable images or data included in this article.

## Author contributions

NFMR, SS, NNINMF, YYX, NCD, and RR contributed to conception and design of the study. NFMR and NNINMF organized the database. NFMR performed the statistical analysis and wrote the first draft of the manuscript. NFMR, NNINMF, and YYX wrote sections of the manuscript. All authors contributed to the manuscript revision, read, and approved the submitted version.
